# Optimization of biogas potential using kinetic models, response surface methodology, and instrumental evidence for biodegradation of tannery fleshings during anaerobic digestion

**DOI:** 10.1515/biol-2022-0721

**Published:** 2023-09-19

**Authors:** Kavan Kumar V., R. Mahendiran, P. Subramanian, S. Karthikeyan, A. Surendrakumar, V. Kumargouda, Ravi Y., Sharda Choudhary, Ravindra Singh, Arvind K. Verma

**Affiliations:** Department of Renewable Energy Engineering, CTAE, MPUAT, Udaipur, Rajasthan, 313001, India; Department of Renewable Energy Engineering, Agricultural Engineering College and Research Institute, TNAU, Coimbatore, Tamil Nadu, 641003, India; Department of Processing and Food Engineering, College of Agricultural Engineering, UAS, GKVK, Bangalore, Karnataka, 560065, India; ICAR-National Research Centre Seed Spices, Ajmer, Rajasthan, 305206, India; Department of Agricultural Engineering, Agricultural Engineering College and Research Institute, TNAU, Coimbatore, Tamil Nadu, 641003, India; Post Harvest Technology Centre, Agricultural Engineering College and Research Institute, TNAU, Coimbatore, Tamil Nadu, 641003, India; Department of Farm Machinery and Power, Agricultural Engineering College and Research Institute, TNAU, Coimbatore, Tamil Nadu, 641003, India

**Keywords:** tannery fleshings, proteolytic enzymes, biogas, kinetics and instrumental analysis

## Abstract

The optimization of the batch size experiment was run for a hydraulic retention time of 45 days using proteolytic enzyme pretreatment. The highest amounts of biogas were produced in comparison to conventional BDS (25:75), which is not processed with enzymes, and there was an increase in the biogas generation of 13.9 and 18.57%. The kinetic models show the goodness of fit between 0.993 and 0.998 and the correlation coefficient’s value domain was [−1, 1] from a statistical perspective. The Box–Behnken design was carried out using the response surface methodology at different levels of independent parameters to optimize the process. Different instruments were evaluated to determine the chemical structure change and the contamination of the different treatments and the raw sample of tannery fleshings was determined. Thermogravimetric analysis was conducted to determine the loss of weight on thermal degradation. The Fourier transform infrared spectrometry was carried out to determine the different functional groups, such as –OH, –CH, –NH, and C–O, present in the samples of tannery fleshings. Scanning electron microscopy and energy dispersive X-ray analysis were carried out to determine the morphological alterations in the substrate, digestate, enzyme-pretreated fleshings, and the chemical composition of samples.

## Graphical abstract

**Figure:**
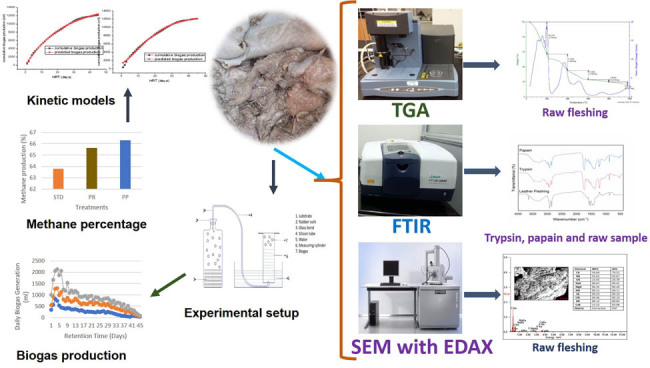

## 1 Introduction

Tannery industries are among the most polluting sectors due to the generation of high solid wastes and wastewater [1]. The production of tannery primary sludge and secondary sludge is enormous, and when tannery enterprises are structured as concentrated districts, tannery wastewater is treated in specialized industrial wastewater treatment plants [2]. Pre-tanning, tanning and crusting, and refinishing procedures are all steps in the multi-step sequential process of tanning leather. Therefore, depending on the production phase they are produced from, tannery solid wastes might vary greatly in terms of quantity and quality. Pre-tanning and tanning activities produce the majority of the pollution burden. The majority of pre-tanning solid wastes consist of fleshing, skin trims, and hair. Due to their high chemical pollution content and the presence of resistant chemicals, tannery fleshings (TF) and tannery primary sludge have historically been managed through landfill disposal and cremation [3]. Anaerobic digestion (AD) has emerged as a viable option for the integrated and sustainable management of tannery wastewater and solid wastes in response to new strict legislation and environmental policies that encourage alternative eco-friendly treatments. Other alternate treatments, such as AD, have been suggested to divert fleshing and other leather wastes from the final landfill disposal or incineration in favour of resource and/or energy recovery [4,5]. Composting, recovering tanning ingredients, producing biodiesel, and producing proteolytic enzymes from fleshing fermentation are a few of the described bioconversion processes. Additionally, TF has been subjected to physicochemical treatments for more than 20 years for the recovery of fat and protein as well as the production of glue, typically in centralized industrial settings where the economies of scale make it affordable to collect and treat TF for material recovery [6]. For instance, a single company (SGS, Pisa, Italy) collects fleshing from roughly 400 tanneries in the Tuscany tannery district, separates the fat and protein fractions, and then markets them to the cosmetic and fertilizer industries, respectively [7]. However, such a treatment necessitates laborious and energy-intensive procedures, and the market for the resulting by-products is subject to significant price swings [8].

Numerous research studies are currently available mentioning tannery districts in India, China, Latin America, and Italy. In general, studies on the AD of leather solid wastes concur that the process is feasible while cautioning against potential operational issues linked to imbalanced C/N ratios and inhibiting conditions from ammonia, long-chain fatty acids, and sulphur dioxide [9,10]. Furthermore, tanning processes try to stabilize the collagen in leather, biological treatments, and in particular AD, proved to perform better for untanned wastes than for tanned ones. The analysis of the anaerobic biodegradability of tannery wastes containing various concentrations of chrome, the lower the methane output, primarily because of the low hydrolysis of tanned stable material, the higher the chrome content. The same authors noted that to avoid low-performance issues, careful selection of seed sludge and substrate hydrolysis pre-treatments is needed. The biodegradability of untanned, chrome-tanned, and vegetable-tanned leather solid wastes was also studied by Damtie et al. [11]. Untanned wastes produced the maximum amount of methane; however, vegetable-tanned wastes were more biodegradable than chrome-tanned ones. The same study also assessed the impact of detanning pretreatment, which led to an improvement in the biodegradability of wastes.

However, the bulk of the research that has been reported on AD has used a combination of tannery wastes and other substrates that came from the tannery industry. The goal of this research is to better understand how AD is used in technology. In the context of its use as an on-site treatment option for decentralized tanneries, of single Tannery Fleshing. Particularly, critical conditions related to the liquefaction of tannery fleshings have been studied in order to define the applicability range of proteolytic enzymes to reduce the size of fleshings for the process of AD. The investigation of sample analysis techniques and equipment to study the changes in the structure, chemical composition, thermal decomposition, and functional groups in the organic substance were estimated using the thermogravimetric analysis (TGA), Fourier transform infrared spectrometry (FTIR), scanning electron microscopy (SEM), and energy dispersive X-ray (EDAX) analysis.

## 2 Materials and methods

### 2.1 Physicochemical properties and instrumental analysis of feedstock and inoculum

The physicochemical properties, such as total solids, volatile solids, total organic carbon, total Kjeldahl nitrogen, and C/N ratio, were estimated using the standard procedures mentioned in the American Public Health Association [12].

### 2.2 Enzymatic pretreatment of tannery fleshings

The application of enzymes in waste treatment using a biotechnological method is a new area of research. The use of enzymes to speed up the digestive process is an option, and the current study uses proteolytic enzymes. Proteolytic enzymes are enzymes that help in the breakdown of proteins [13]. These enzymes are extracted from animals, bacteria, plants, and fungi. Some of the proteolytic enzymes may be found in supplements like ficin, papain, trypsin, chymotrypsin, and trypsin. In this study, trypsin and papain enzymes were selected at 5U for the treatment of 1 kg fleshings.

#### 2.2.1 Trypsin enzyme pretreatment

Trypsin is one of the three major types of industrial enzymes, with applications in the leather industry, food processing, and bioremediation. A protease enzyme catalyses the breakdown of proteins into smaller polypeptides or single amino acids. Hydrolysis, a reaction in which water breaks the bonds, is used to disrupt the peptide links within the proteins.

#### 2.2.2 Papain enzyme pretreatment

Papain enzyme is obtained from the papaya fruit. The papain enzyme is also called papaya proteinase I and has a broad pH of 5–7.5 and a temperature stability of 70–90°C. It is very popular in various applications. It is used as a meat tenderizer; the enzyme makes its way into muscle and hydrolyses primarily connective protein tissues (collagen) and softens the muscle. Papain has to be used in a low quantity to prevent the high liquefaction of the substrates.

#### 2.2.3 Batch-scale experiment for pretreated tannery fleshings

**Figure 1:**
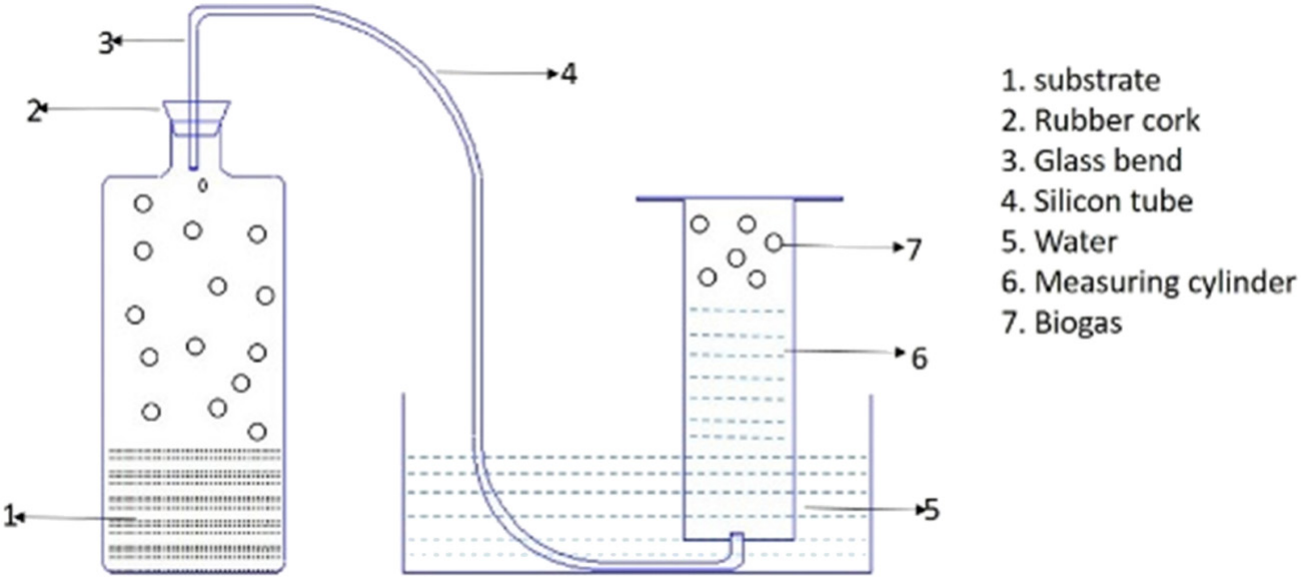
Water displacement set-up for biogas measurement.

The study on biomethane potential using batch-scale reactors was carried out with the best treatment selected from the batch experiment. Another study was carried out after the treatment of tannery fleshings with 82.5 IU of proteolytic enzymes with a feeding bio-digested slurry and tannery fleshings in the proportion of 0.25:0.75 with three treatments. The first treatment was performed without enzyme addition, and the other two treatments were performed using trypsin and papain enzymes. After feeding substrate and inoculum into the reactors, the reactors were closed and provided with a gas outlet. Duplicate reactors were operated to find the repeatability, and the performance was reported. The daily biogas production was measured using the water displacement method, as shown in Figure 1, and the methane content was estimated using a 5% alkali solution.

### 2.3 Kinetic studies for biogas production

Kinetic analysis is a commonly used concept for identifying the significance of inter-variable interactions in order to guide experimental design, evaluate experimental results, and describe particular system performance. Experimental kinetic studies can be used to simulate digester behaviour and forecast the biogas output of a running plant under similar conditions. To forecast biogas output and evaluate kinetic parameters, a first-order model and modified Gompertz models were used in this work. Using IBM SPSS software 25.0, the experimental cumulative biogas was utilized to estimate the parameters using non-linear regression.

#### 2.3.1 First-order kinetic model

The following first-order kinetics equation is used to predict the biogas yield production:

(1) \[P={P}_{0}{[}1-\exp (-k\times t)],]\]

where *P* is the cumulative biogas yield, *P*_0_ is the ultimate biogas yield, *k* is the first-order rate constant, and *t* is the time. The first-order kinetics used an empirical linear regression to determine the rate of reaction, where the value of the slope of the linear plot represents the given substrate characteristics. However, the first-order model’s linear form, which is an exponential form, cannot be used to appropriately account for and predict cumulative biogas production during the entire process, particularly after the exponential phase.

#### 2.3.2 Modified Gompertz model

The modified Gompertz model is a non-linear kinetic model, which is used to calculate the length of the lag phase and biogas production rate:

(2) \[p={P}_{0}\times \exp \left\{-\exp \left[\frac{R\times 2.7183}{{P}_{0}}(L-t)+1\right]\right\},]\]

where *L* is the lag phase duration, *R* is the biogas production rate, and *P*_0_ is the biogas potential at time *t*. The standard statistical metric is used to study the model performance. The root mean square error (RMSE) is calculated for the actual and predicted biogas production values using the following equation:

(3) \[{\mathrm{RMSE}}={\left[\frac{1}{m}\mathop{\sum }\limits_{j=1}^{m}{\left(\frac{{{\mathrm{d}}}_{j}}{{y}_{j}}\right)}^{2}\right]}^{\frac{1}{2}},]\]

where *m* is the number of data pairs, *d* is the difference between experimental and predicted yield, and *Y* is the measured biogas yield.

#### 2.3.3 Correlation studies for model parameters

The correlation coefficients for different parameters of first-order and modified Gompertz kinetic models were studied using ORIGIN 2019 software. The results are elaborated with the proper justification in the following headings of the research work.

#### 2.3.4 Optimization of parameters for biogas production

Response surface methodology (RSM) is a significant statistical approach for the investigation of complicated processes. As a result, these techniques were used to optimize the process parameters for the production of biogas from the proteolytic pretreated tannery fleshings. RSM is a combination of statistical approaches for conducting experiments, developing models, assessing the impacts of variables, and locating optimal conditions for desired responses [14]. The RSM analysis involves fitting the experimental values of biogas production to a standard equation and then optimizing the value using appropriate optimization tools or mathematical solutions. The process parameters such as pH, temperature, and hydraulic retention time (HRT) were optimized for maximum biogas production.

#### 2.3.5 Design of experiments

The RSM deals with finding the most optimal/desired parameters for an experiment. To design the experiment, the Box–Behnken design (BBD) was used, and the design consisted of three variables and three levels including the 17 experiments formed by 5 central points [15]. Three independent variables considered to influence biogas production are pH, temperature, and HRT, as shown in Table 1. This shows the RSM experimental design of independent parameters and layout for these three levels and three variables. During the experiment, all of these variables and responses were properly measured and evaluated experimentally. The RSM for BBD in the experimental data was analysed using the package of design expert version 12.0 software.

**Table 1:** Levels of input values for BMP studies of tannery fleshings

Variables	Coded	Range and levels		
		Low level (−1)	Centre level (0)	High level (1)
pH	X1	7	7.5	8
Temperature (°C)	X2	28	32	36
HRT (days)	X3	1	23	45

### 2.4 Characterization of substrate and digestate

Tannery fleshings were characterized before the experimental trials and the obtained digestate was also characterized using different analytical instruments to check the parametrical change in the output. The procedures adopted are briefly mentioned. The samples were also characterized using TGA, FT-IR analysis, SEM analysis, and EDAX analysis [16]. The samples were pretreated using the proteolytic (trypsin and papain) enzymes to reduce the time of hydrolysis in the AD process.

#### 2.4.1 TGA

TGA is a technique used to estimate the thermal stability of organic materials and volatile component fractions by considering that the weight change occurs in a sample with a constant rate of heating. About 8.6920 g of raw lime and 7.3230 g of delimed tannery fleshing samples were analysed to determine the changes occurring in the thermal process. The Ramp method used in TGA Q50 V20.13 Build 39 with N_2_ as an inert gas with a flow rate of 100 mL/min [17].

#### 2.4.2 FT-IR analysis

Substrate, digestate, and proteolytic enzyme-treated samples were air-dried to remove moisture. The dried sample pellets were made in a 5:1 ratio and subjected to FT-IR analysis using a transmission mode [18]. The measurements were carried out in the mid-infrared range wavenumber between 4,000 and 500 cm^−1^. The measurement information of FTIR settings is given in Table 1.

#### 2.4.3 SEM and EDAX analysis

The air-dried samples were coated with gold in an argon medium. SEM and EDAX analyses were carried out on a scanning device attached to a QUANTA 250 Everhart Thornley Detector electron microscope at 8 kV, accelerated with an electron beam [19]. The digestion of the substrate during the AD is determined by SEM analysis, and the compounds in the sample are determined by the EDAX analysis.

## 3 Results and discussion

The results obtained from the experiments carried out are discussed in this section. The physicochemical properties of tannery fleshings and inoculum, daily biogas production, methane percentage, and bio-digestate values are presented.

### 3.1 Physicochemical properties of the feedstock and inoculum

The estimated physical and chemical parameters for tannery fleshings and the inoculum as bio-digested slurry are given in Table 2.

**Table 2:** Physicochemical properties of fleshings, cow dung, elephant dung, and bio-digested slurry

Sl. No.	Parameter	Tannery fleshings	Bio-digested slurry
1	pH	11.0–12.0*	7.0–8.0*
2	Moisture content (%)	82.1 ± 0.20*	79.24 ± 0.78*
3	Total solids (%)	17.9 ± 0.20*	20.76 ± 0.78*
4	Volatile solids (%)	66 41 ± 1.44*	69.93 ± 0.86*
5	Total organic carbon (g/kg)	0.95 ± 0.01*	19.7 ± 0.34*
6	Total Kjeldahl nitrogen (g/kg)	36.0 ± 2.1*	1.09 ± 0.71*
7	C/N ratio	9.3 ± 0.55*	18.07 ± 0.48*

*Values are expressed as mean ± SD on a dry basis.

### 3.2 Batch AD of pretreated tannery fleshings

**Figure 2:**
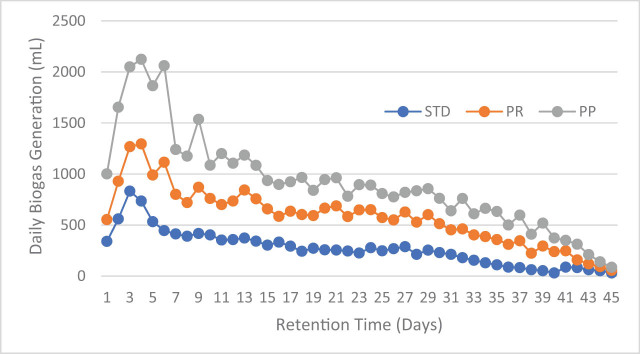
Daily biogas generation of the enzymatic pretreated tannery fleshings.

**Figure 3:**
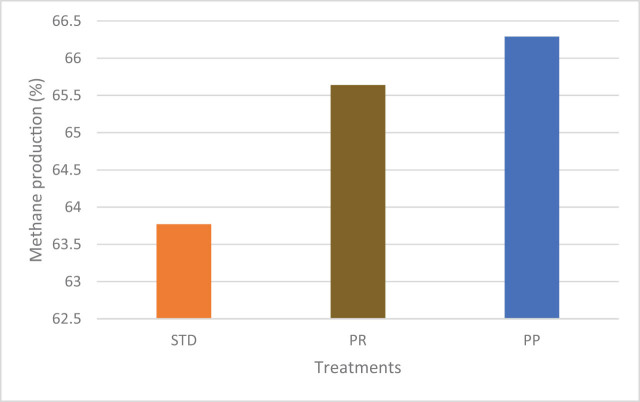
Comparative methane content of the pretreated tannery fleshing treatments.

The pretreatment helps to exploit the substrate for better biogas production. The best treatment was selected from a previous study, and an additional biomethane potential experiment was carried out for the tannery fleshings pretreated with trypsin and papain enzyme. The disintegrated tannery fleshings were collected and subjected to the batch scale AD experiment for a retention period of 45 days. The daily gas measurement was observed using the water displacement method and methane content was estimated. The gas production and methane content are presented in Figures 2 and 3.

Maximum biogas of 12,118 mL from the standard BDS (25:75), 14073.25 mL from the trypsin-treated BDS (25:75), and 14881.25 mL from papain-treated BDS (25:75) were generated with methane contents of 63.77, 65.64, and 66.29%. There is an increase in the biogas production of 13.9% for trypsin and 18.57% for papain treatment compared to standard BDS (25:75) without pretreatment.

### 3.3 Simulation of experimental data using first-order kinetics and modified Gompertz model and correlation coefficient

**Figure 4:**
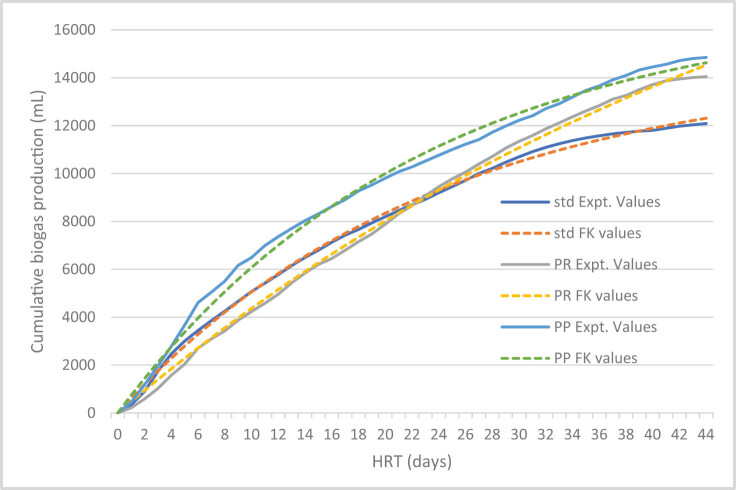
Simulation values for experimental and predicted values of biogas production from the first-order kinetic model.

**Figure 5:**
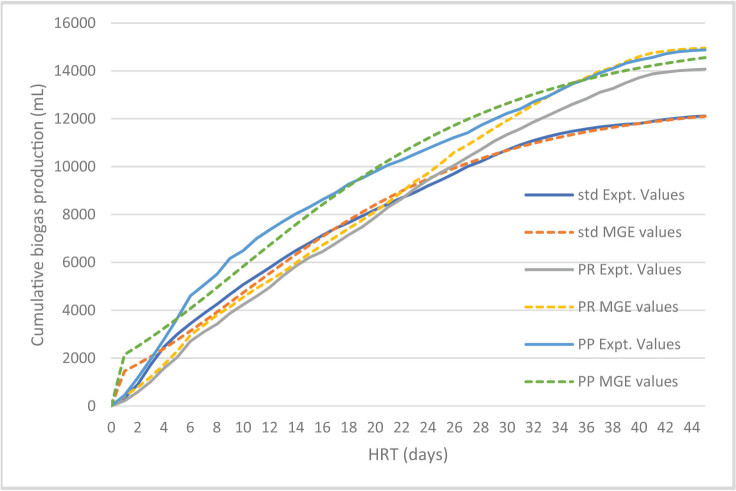
Simulation values for experimental and predicted values of biogas production from the modified Gompertz model.

In this section, we predicted the biogas production rate using the kinetic models. The simulated experimental and predicted values are shown in Figures 4 and 5, whereas the goodness of fit for the first-order kinetic model ranges from 0.994 to 0.998 and the goodness of fit for the modified Gompertz model ranges from 0.983 to 0.993. The different parameters obtained from the kinetic model studies are presented in Table 3.

**Table 3:** Simulation values for experimental and predicted values of biogas production from the first-order kinetic model

Sl. no.	Parameter	Standard	Trypsin	Papain
**First-order kinetic model**				
1.	*C* – Experimental (mL)	12,118	14073.25	14881.25
2.	*C* – Predicted (mL)	12399.56	14745.20	14735.53
3.	*P*_0_ (mL/day)	402.27	412.73	439.82
4.	*t* (days)	0.043	0.018	0.044
5.	Residual sum of squares	1087103.81	2208444.25	4233332.71
6.	Corrected sum of squares	525677884.2	825190107.8	716,421604
7.	*R* ^2^	0.998	0.917	0.994
8.	RMSE	2.80	3.97	1.74
**Modified Gompertz model**				
1.	*C* – Experimental (mL)	12,118	14073.25	14881.25
2.	*C* – Predicted (mL)	12102.60	14945.8	14558.25
3.	*R* (mL/day)	407.70	433.04	446.44
4.	*t* (days)	1.615	2.057	3.071
5.	Residual sum of squares	3579366.18	5892716.24	12301598.6
6.	Corrected sum of squares	525677884.2	825190107.8	716,421604
7.	*R* ^2^	0.993	0.992	0.983
8.	RMSE	1.04	1.94	3.42

As shown in Table 3, the daily biogas production potential ranges from 402.27 to 439.82 mL/g VS for the first-order kinetic model and 407.70 to 446.44 mL/g VS for the modified Gompertz model.

### 3.4 Correlation studies for kinetic model parameters

The relation between the different parameters of kinetic model parameters using first-order and modified Gompertz models are shown in Tables 4 and 5.

**Table 4:** Correlation coefficients of first-order kinetic model parameters

Correlation coefficient	*G*	*K*
**Standard samples**		
*G*	1.000	−0.966
*K*	−0.966	1.000
**Trypsin enzyme sample**		
*G*	1.000	−0.996
*K*	−0.996	1.000
**Papain enzyme sample**		
*G*	1.000	−0.963
*K*	−0.963	1.000

**Table 5:** Correlation coefficient of the modified Gompertz kinetic model parameters

Correlation coefficient	*P*	*R*	*L*
**Standard samples**			
*P*	1.000	−0.714	0.903
*R*	−0.714	1.000	−0.537
*L*	0.903	−0.537	1.000
**Trypsin enzyme sample**			
*P*	1.000	−0.843	0.814
*R*	−0.843	1.000	−0.732
*L*	0.814	−0.732	1.000
**Papain enzyme sample**			
*P*	1.000	−0.749	−0.582
*R*	−0.749	1.000	0.915
*L*	−0.582	0.915	1.000

The correlation coefficient’s value domain is [−1, 1] from a statistical perspective. Many other investigations have confirmed it, in addition to our own. In addition to highlighting the linear relationship between two data sets in a space of observation objects, the correlation coefficient also highlights the variability of two data sets, making this value domain more important than [0, 1]. Two datasets might exhibit the same pattern or a downward trend (in the case of positive correlation). Additionally, the first data set might be increased while the second is decreased; conversely, in the event of a negative correlation, the first data set could be decreased while the second is increased.

### 3.5 Optimization of the BMP experiment for tannery fleshings

One of the most crucial factors to be considered in an anaerobic digester is the precise anticipation of the volume of producible biogas. The potential biogas yield is a function of the input factors and depends on the chemical makeup of the feedstock. The influence of the input variables on the biogas yield is expressed here in the simplified form of the coded and actual values of the independent variables [20]. The positive and negative signals placed in front of each model phrase show both a complementary and a competitive impact on the reaction.

#### 3.5.1 Analysis of variance (ANOVA)

The model *F*-values imply that the model is significant. There is only a 0.01% chance that this large *F*-value could occur due to noise. *p*-values <0.0500 indicate that the model terms are significant; this was also reported by some researchers [21]. In this case, *C* is a significant model term. Values greater than 0.1000 indicate that the model terms are not significant. If there are many insignificant model terms (not counting those required to support hierarchy), model reduction may improve the model. The ANOVA values of the models are shown in Tables 6–8.

**Table 6:** ANOVA for the standard sample of tannery fleshings

Source	Sum of squares	df	Mean square	*F*-value	*p*-value	
**Model**	1.919 × 10^5^	3	63963.38	121.94	<0.0001	Significant
*A* – pH	0.0000	1	0.0000	0.0000	1.0000	
*B* – Temperature	0.0000	1	0.0000	0.0000	1.0000	
*C* – HRT	1.919 × 10^5^	1	1.919 × 10^5^	365.83	<0.0001	
**Residual**	6818.89	13	524.53			
Lack of fit	6818.89	9	757.65			
Pure error	0.0000	4	0.0000			
**Cor total**	1.987 × 10^5^	16				

**Table 7:** ANOVA for trypsin-pretreated tannery fleshings

Source	Sum of squares	df	Mean square	*F*-value	*p*-value	
**Model**	2.211 × 10^5^	3	73704.17	228.00	<0.0001	Significant
*A* – pH	0.0000	1	0.0000	0.0000	1.0000	
*B* – Temperature	0.0000	1	0.0000	0.0000	1.0000	
*C* – HRT	2.211 × 10^5^	1	2.211 × 10^5^	683.99	<0.0001	
**Residual**	4202.47	13	323.27			
Lack of fit	4202.47	9	466.94			
Pure error	0.0000	4	0.0000			
**Cor total**	2.253 × 10^5^	16				

**Table 8:** ANOVA for papain-pretreated tannery fleshings

Source	Sum of squares	df	Mean square	*F*-value	*p*-value	
**Model**	3.482 × 10^5^	3	1.161 × 10^5^	14990.36	<0.0001	Significant
*A* – pH	0.0000	1	0.0000	0.0000	1.0000	
*B* – Temperature	0.0000	1	0.0000	0.0000	1.0000	
*C* – HRT	3.482 × 10^5^	1	3.482 × 10^5^	44971.07	<0.0001	
**Residual**	100.65	13	7.74			
Lack of fit	100.65	9	11.18			
Pure error	0.0000	4	0.0000			
**Cor total**	3.483 × 10^5^	16				

**Figure 6:**
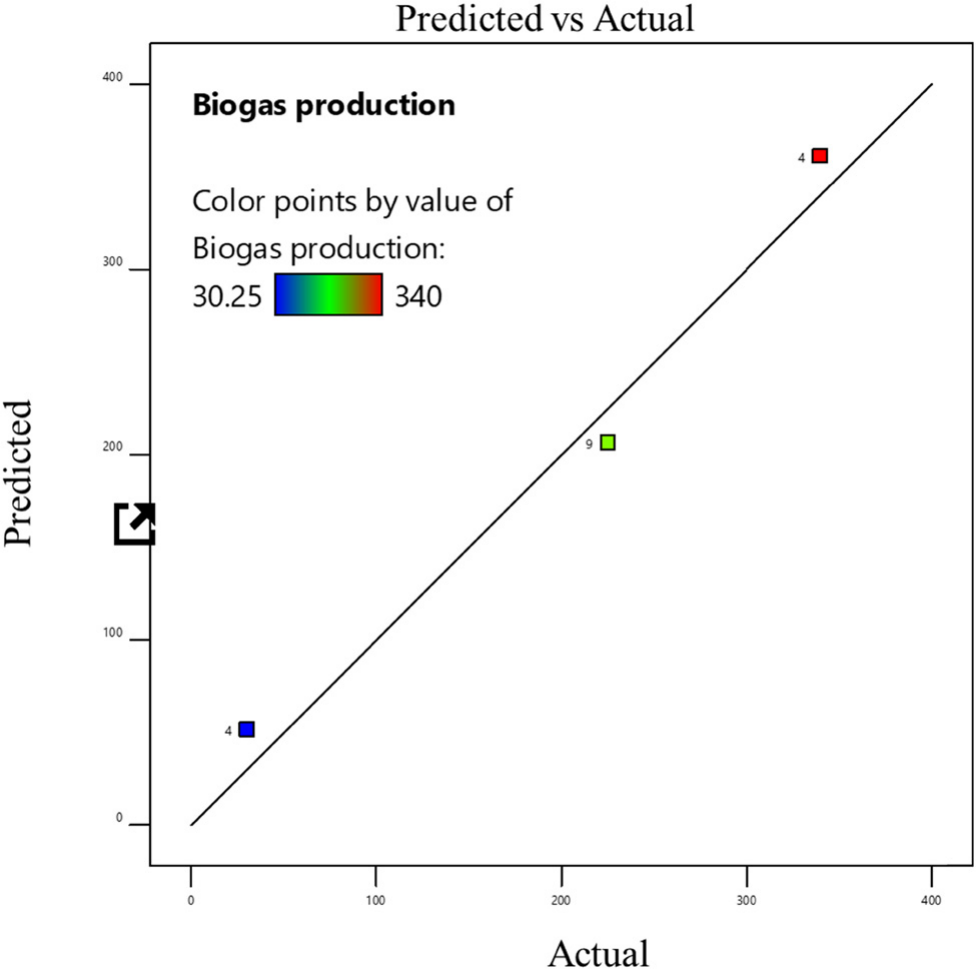
Actual and predicted values of biogas production from the standard sample of tannery fleshings.

**Figure 7:**
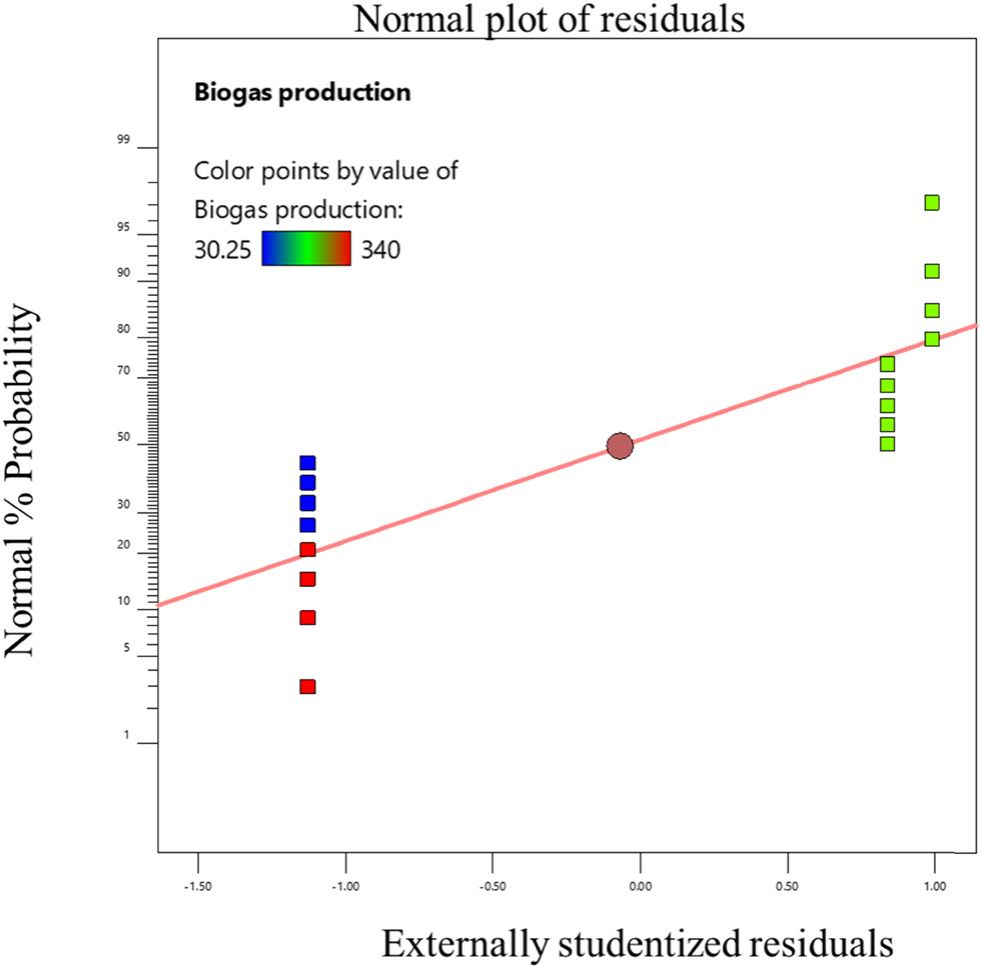
Normal probability plot for the standard sample of tannery fleshings.

**Figure 8:**
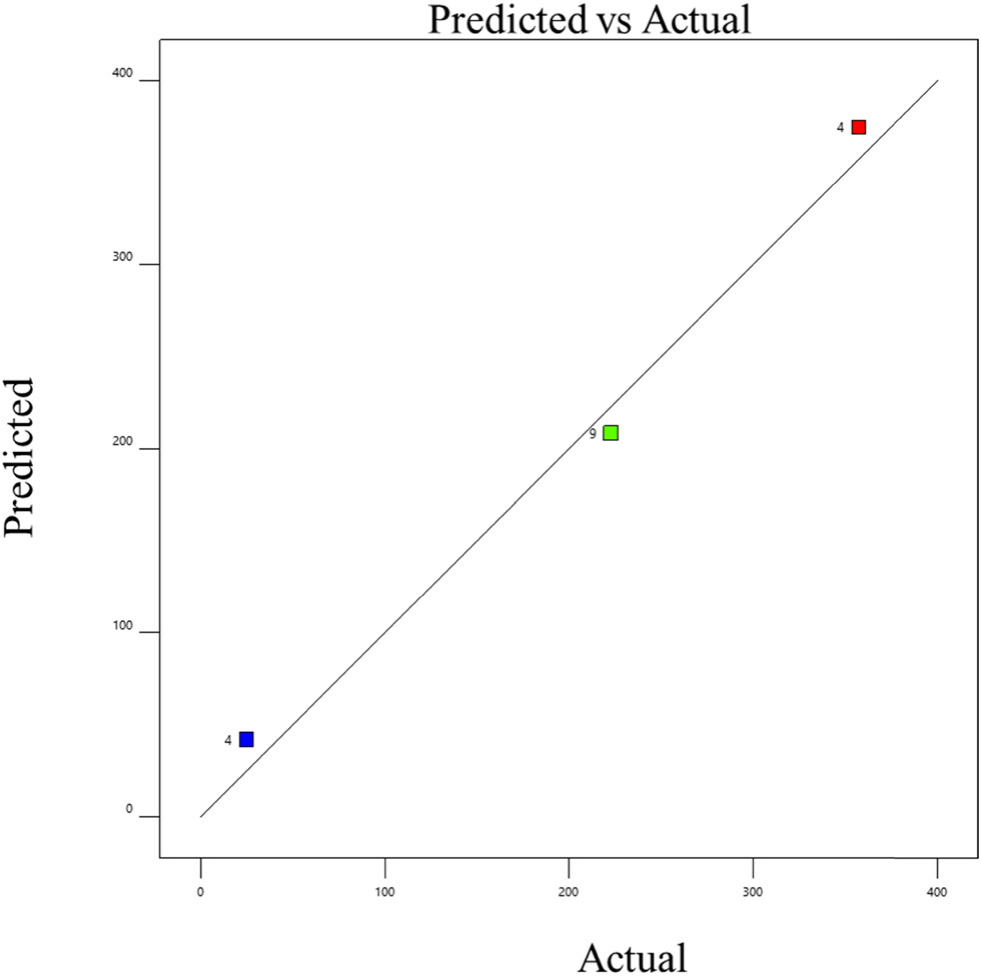
Actual and predicted values of biogas production from trypsin-pretreated tannery fleshings.

**Figure 9:**
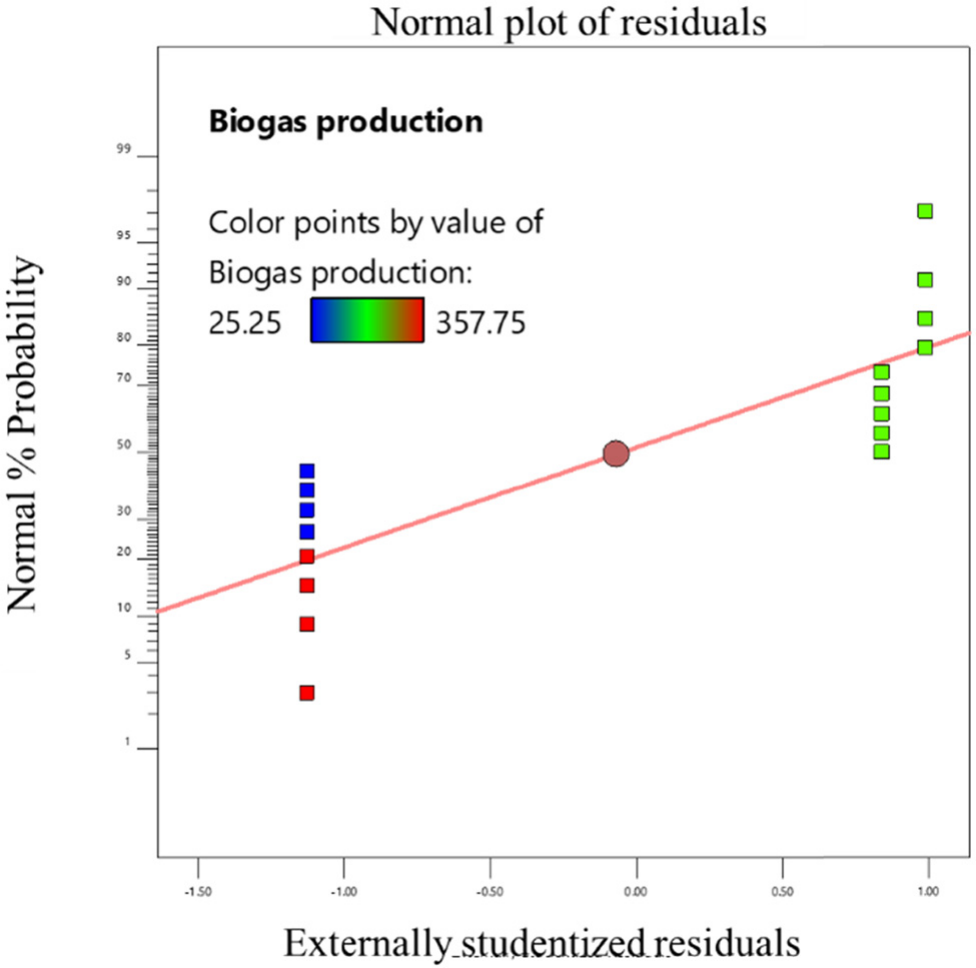
Normal probability plot for trypsin-pretreated tannery fleshings.

**Figure 10:**
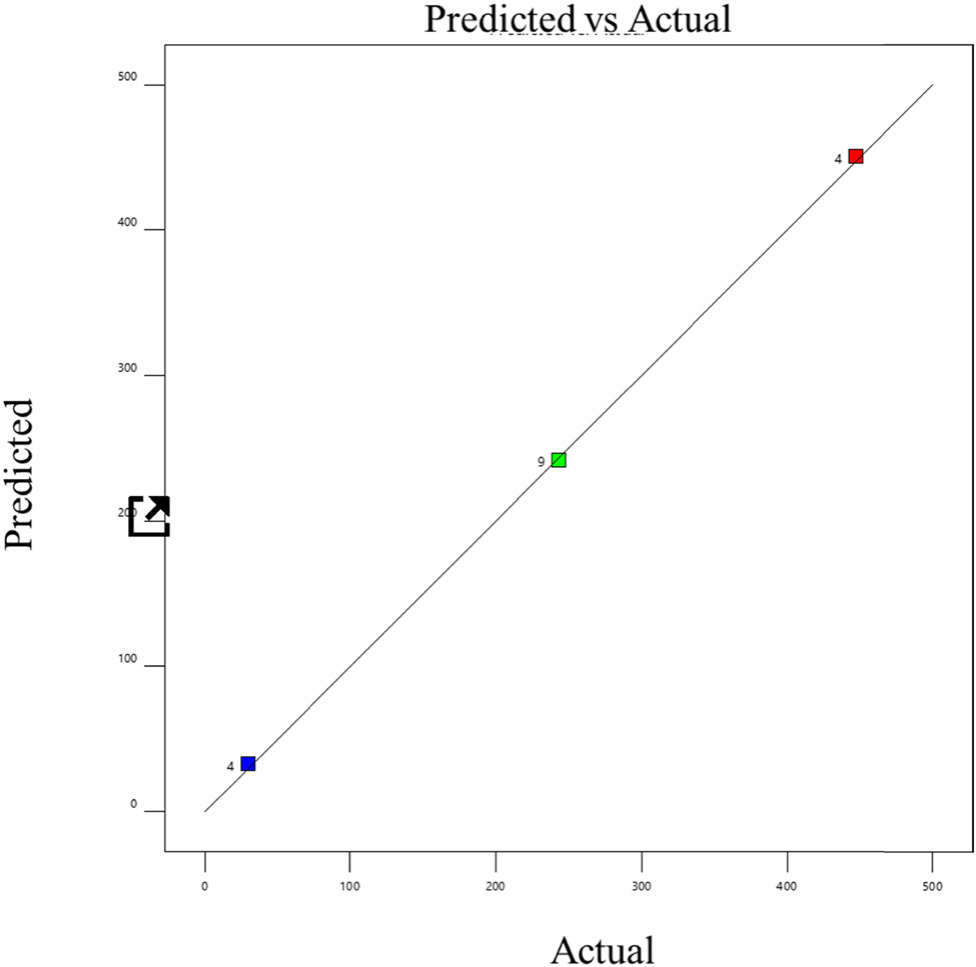
Actual and predicted values of biogas production from papain-pretreated tannery fleshings.

**Figure 11:**
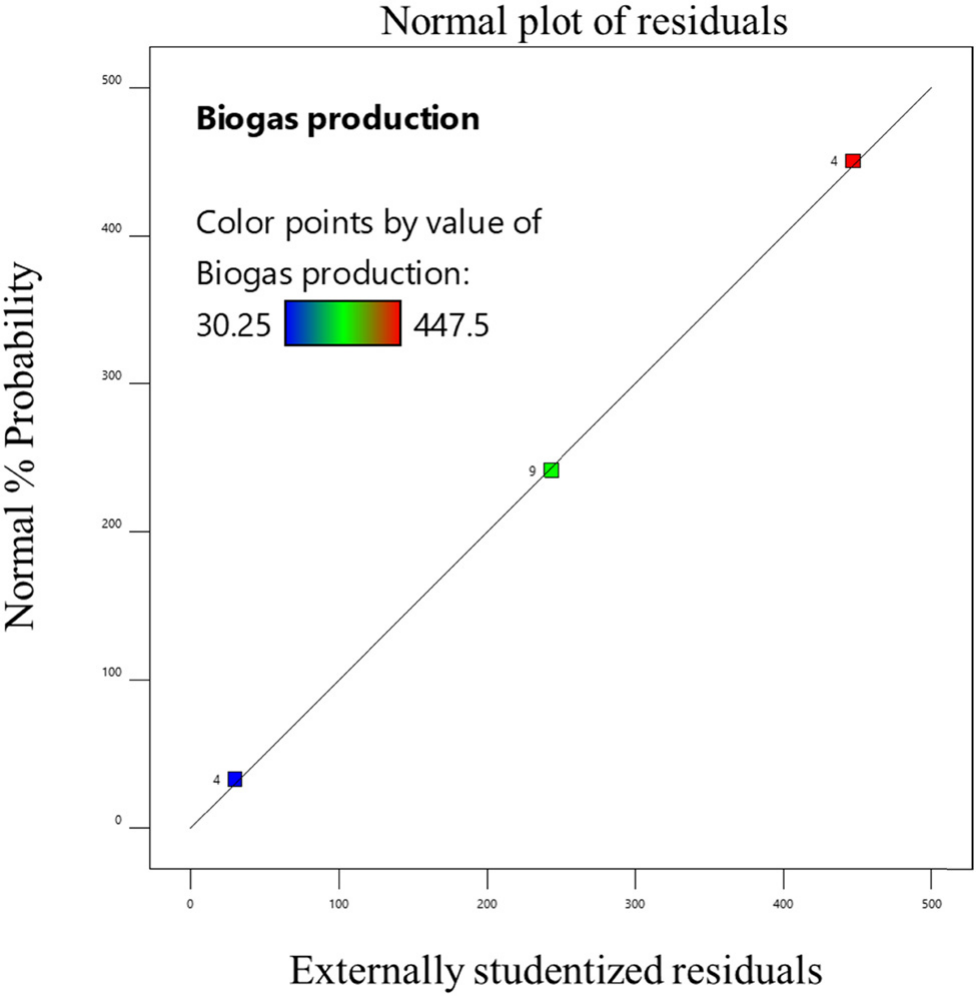
Normal probability plot for papain-pretreated tannery fleshings.

The experimental data were fitted using a cubic model, and the statistical significance for linear terms for biogas generation was calculated as indicated in the tables. By using the least-squares method, the *R* ^2^ value ranged from 0.9368 to 0.996, indicating a satisfactory fit between the model and the data. The model’s *F* values indicate that it is significant (*P* 0.0001), according to the data. Significant linear terms are present (*P* 0.0001). The developed model was sufficient for forecasting the response because the lack of fit *F* value was non-significant [22]. This demonstrated that the model did not contain the non-significant terms. Consequently, one may utilize this model to explore the design space [23]. The probability and the actual vs predicted values of the optimization are shown in Figures 6–11. The biogas production equations for different treatments are as follows:

Biogas production = 368.28 + 7.364*A* + 9.447*B* – 7.04*C* (standard sample).

Biogas production = 381.98 – 3.605*A* – 4.97*B* – 7.55*C* (trypsin-pretreated sample).

Biogas production = 459.56 + 6.238*A* + 8.148B – 9.482*C* (papain-pretreated sample).

### 3.6 Digestate characteristics analysis of pretreated fleshings

At the end of digestion, once the gas production ceases, the digestate was collected from the batch scale experiment and analysed for the total solid reduction, volatile solid reduction, volatile fatty acids, and alkalinity of the tannery fleshings. The results are given in Table 9.

**Table 9:** Performance data of the biomethane potential for the pretreated substrate of 45 days HRT

Parameter	Standard	Trypsin	Papain
VS in the feedstock (g)	11.37	11.37	11.37
I/S ratio	0.33	0.33	0.33
Total biogas generated (mL)	12,118	14073.25	14881.25
Biogas generated per gram of VS added (mg/L)	1065.78	1237.75	1308.82
Methane volume (mL)	7727.64	9279.9	9864.78
Methane content (%)	63.77	65.64	66.29
Methane yield per gram of VS added (mL/g)	679.65	816.17	867.61
Specific CH_4_ production rate (mL CH_4_/g VS/day)	28.31	34.00	36.15
TS reduction (%)	47.53	51.28	52.41
VS reduction (%)	38.84	38.91	39.93
Volatile fatty acids (mg/L)	800	740	690
Alkalinity (mg/L)	4,500	3,700	2,900
VFA/alkalinity ratio	0.17	0.2	0.23

The biogas and methane content of 14881.25 mL was observed from the papain-treated BDS (25:75) with a gas production at 1308.82 mL/g of VS added. The total gas production of 14073.25 mL was found from trypsin-treated BDS (25:75) at 1237.75 mL/g VS and a methane content of 9279.9 at 816.17 mL/g of VS for trypsin and 9864.78 mL and 867.61 mL/g of VS added for papain treatment. A total solid reduction of 51.28 and 52.41% and volatile solid reduction of 38.91 and 39.93% were reported. It was observed that the alkalinity was in the range of 2,900–4,500 mg/L considering all the reactors. The volatile fatty acids were in the range of 690–800 mg/L and well within 500–3,000 mg/L, as mentioned by Sri Bala Kameshwari et al. [24].

### 3.7 Instrumental evidence of the tannery fleshings

The analysis of the samples was carried out by TGA for thermal degradation of the sample, SEM for the identification of structure, and FT-IR spectrometry for the identification of functional groups.

#### 3.7.1 TGA

**Figure 12:**
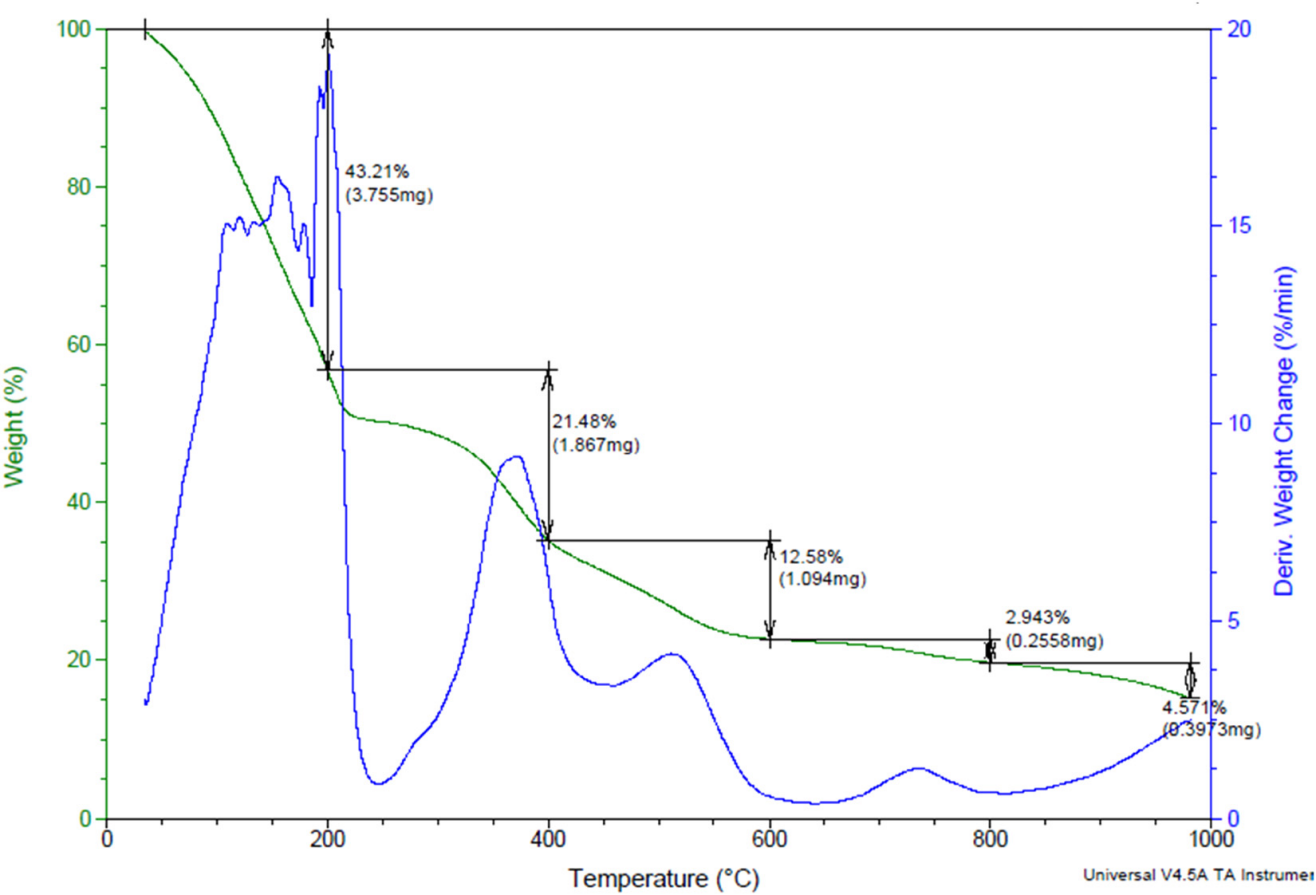
TGA of the raw tannery fleshing sample.

**Figure 13:**
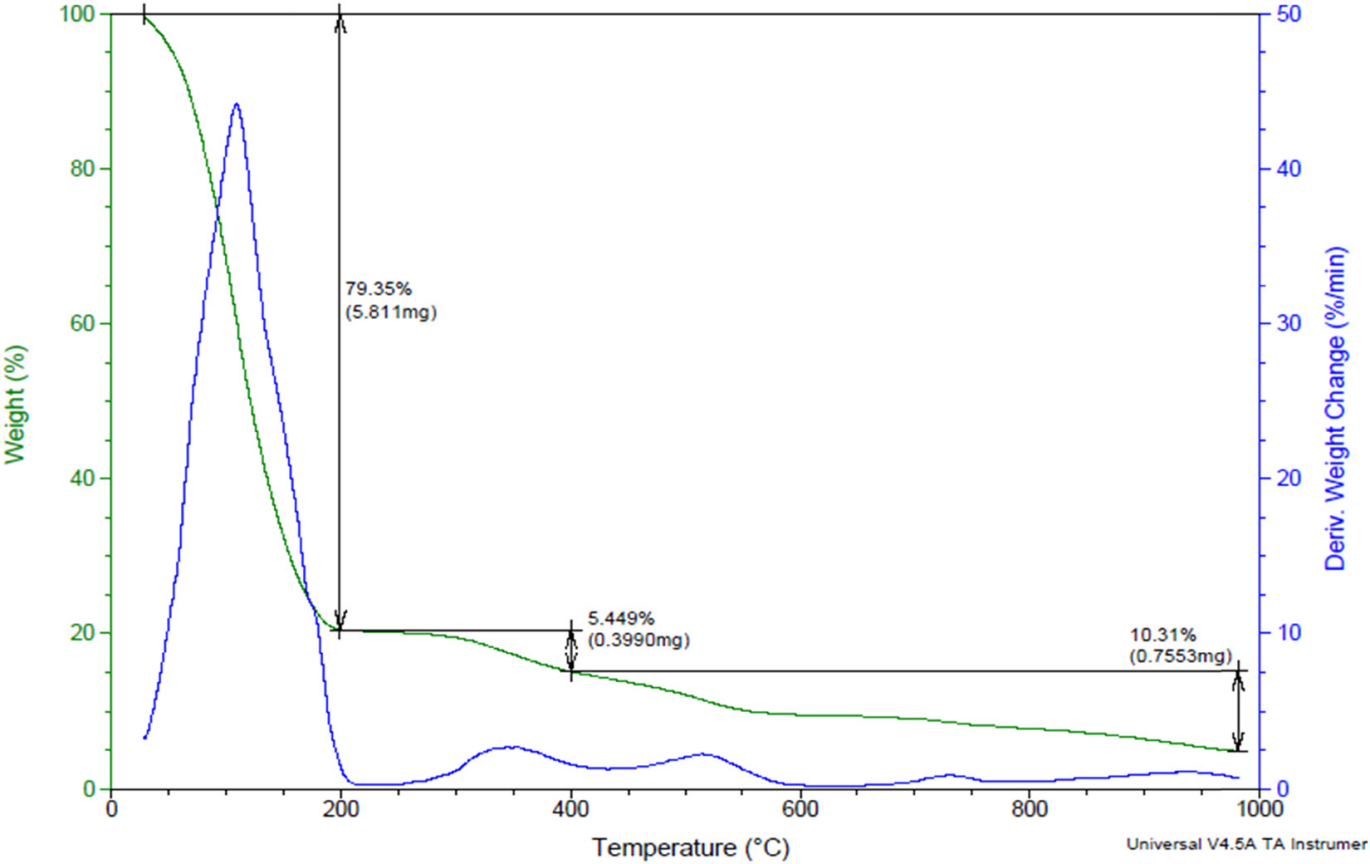
TGA of the treated tannery fleshing sample.

The selected two samples, tannery raw fleshing and treated fleshing samples, show different patterns of weight loss with thermal treatment, as shown in Figures 12 and 13. The loss of moisture content was observed to increase from the ambient to 1,000°C. The TG and DTG curves of the analysis show the respective stages of biomass degradation during the TGA. The TGA of tannery fleshings shows three different phases, namely: moisture removal, devolatization, and biochar formation [25].

The initial moisture removal is observed from ambient temperature to 140°C; cellulose and hemicellulose degradation occurs from 160 to 380°C and the biochar formation occurs from 450 to 600°C. At temperatures of 600–800°C, a peak is observed due to the presence of some volatile compounds in the raw limed tannery fleshings [26]. As shown in Figure 12, the thermal degradation of the raw tannery fleshing occurs in five steps with weight decrease percentages of 43.21, 21.48, 12.58, 2.943, and 4.571%. As shown in Figure 13, the thermal degradation of the treated tannery fleshing occurs in three steps with weight decrease percentages of 79.35, 5.449, and 10.31%, where the thermal degradation percentage of the treated sample is more than that of the raw sample. Similar results were obtained in the work conducted by Fathima et al. [27].

#### 3.7.2 FT-IR analysis

**Figure 14:**
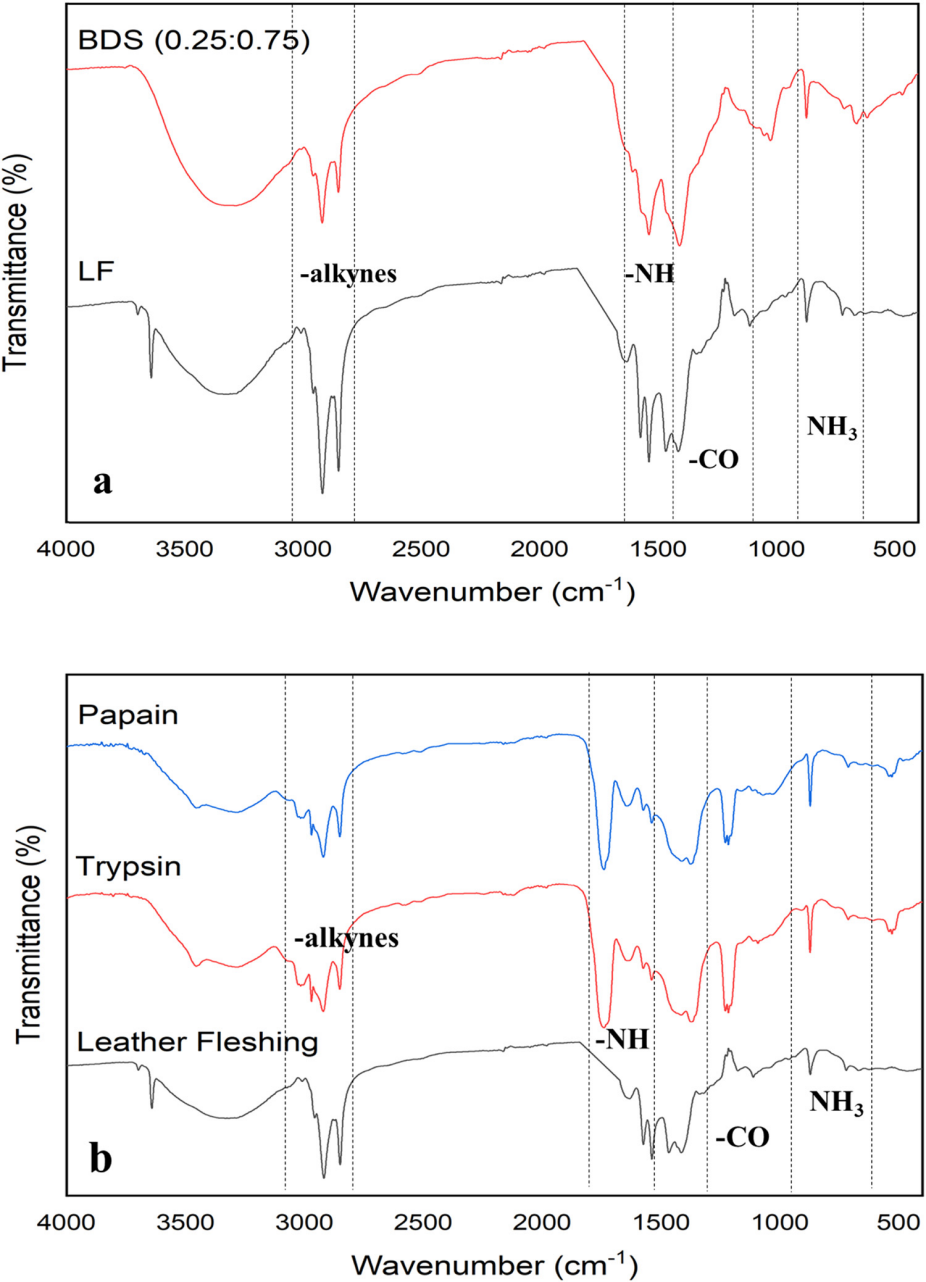
Comparison of FT-IR spectra for the (a) substrate and digestate, and (b) substrate, trypsin, and papain treatment.

FT-IR analysis was performed for the substrate, digestate, and enzyme pretreatment samples. The functional groups of substances contained in the samples were identified using the FT-IR spectra. The spectra of the samples are shown in Figure 14(a) and (b).

Due to the overlap of –OH, –CH, and –NH stretching in FT-IR spectra, a large envelope from 4,000 to 400 cm^−1^ is observed. The FTIR spectrum of leather fleshing (delimed, digestate, proteolytic treated) sample showed –OH stretching of protein molecules in the wavelength between 3,700 and3,600 cm^−1^ but it was not identified in other treated samples of fleshings [28]. The asymmetric and symmetric peaks between 1,300 and 1,080 cm^−1^ were assigned to C–O by the presence of carboxylic acids, esters, and ethers. In-plane bending peaks detected from 1,680 to 1,620 cm^−1^, attributed to C–H due to the presence of hydrocarbons, confirm the occurrence of –CH stretching (hexane, propane). In-plane bending peaks detected at 1,660–1,350 cm^−1,^ attributed to N–H due to the presence of amines, confirm the presence of –NH stretching [29]. The presence of alkynes is shown by the peaks at 2,140–2,100 cm^−1^. The strong N–O bond at 900–700 cm^−1^ and plane bending of ammonia at 1,290–1,090 cm^−1^ indicated the presence of nitro compounds. Similar results of FTIR were reported by Devaraj et al. [30] for co-digestion of tannery solid wastes by the optimization of mix proportions.

The treatment of fleshings with proteolytic enzymes shows the notable changes in the structure. Minor alterations were observed in the amine groups. The conversion of amide bands to amine groups followed by the formation of new acid groups was observed in the FTIR analysis as well as the biodegradation of ketone and –C═O groups to new group of acids was observed [31]. The degradations of the functional groups are helpful in the production of amino acid groups to cut down the hydrolysis process during the AD of tannery fleshings to produce the biogas [32].

#### 3.7.3 SEM analysis

**Figure 15:**
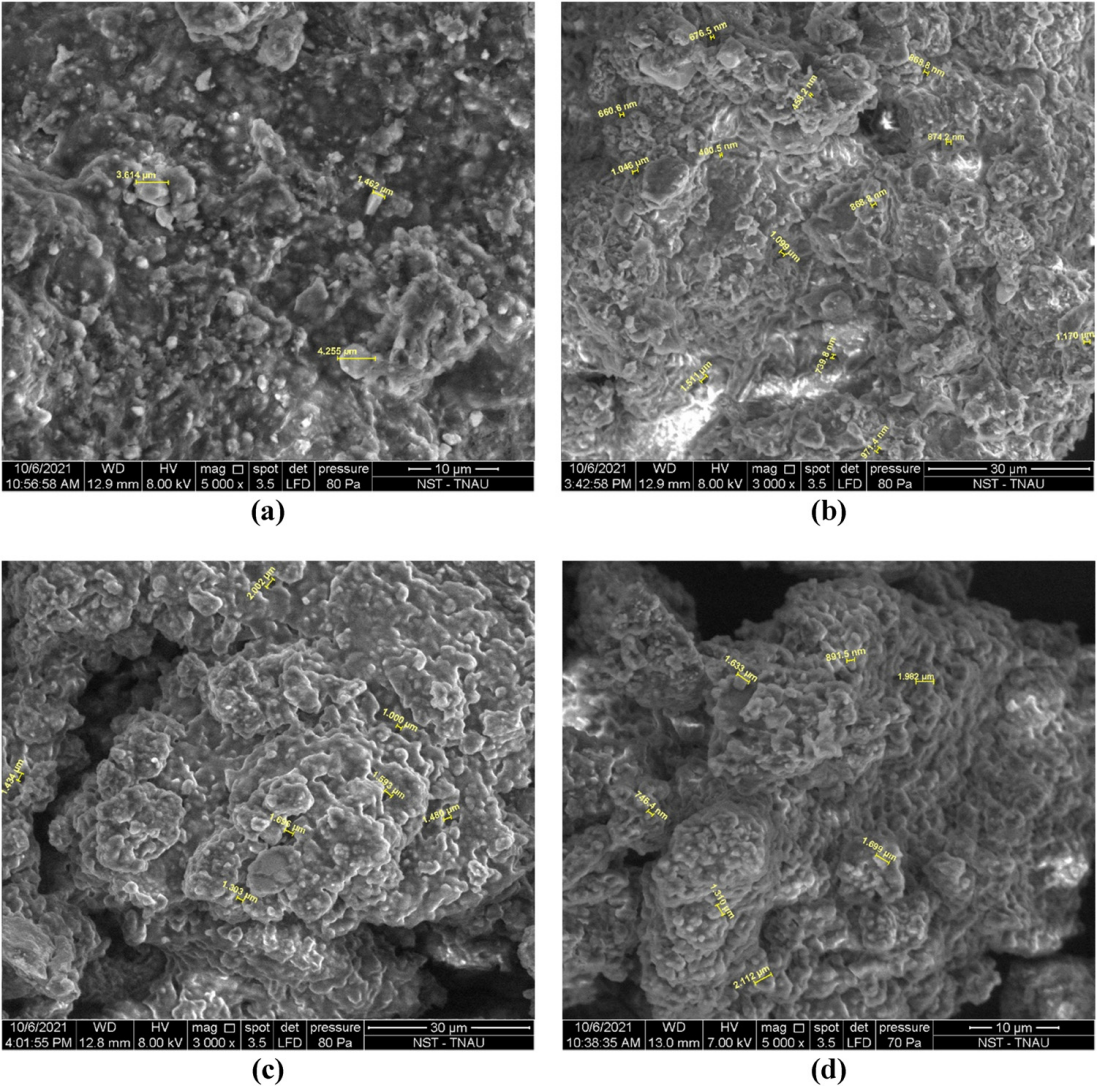
SEM images of the (a) substrate, (b) digestate, (c) trypsin, and (d) papain enzymatic treatment.

The morphological alterations in the substrate, digestate, and enzyme-pretreated fleshings were investigated by SEM. The SEM images are shown in Figure 15(a)–(d). The SEM image reveals the presence of tiny fibrous tissue dispersion in the substrate. Fibrous tissues are connected to the protein matrix. The fibrous protein was digested, as seen by the SEM image of the digestate and the disintegration occurred in the pretreated samples. The digestate has a rough and uneven surface with voids, which could be attributable to the emission of biogas during the digestion process [33].

In addition, the SEM images of the digestate reveal the presence of various-sized particles, which is consistent with the particle size analyses. The visual changes in the tannery fleshing SEM samples confirm the activity of protein hydrolysis during the pretreatment using proteolytic enzymes. There is a notable shrinkage and disintegration of the structure observed at an initial level. The biodegradation of the protein during the pretreatment resulted in the change in the structure of tissues which are even visualized through the SEM images. Similar works on SEM were carried out by other researchers for protein hydrolysis [34].

**Figure 16:**
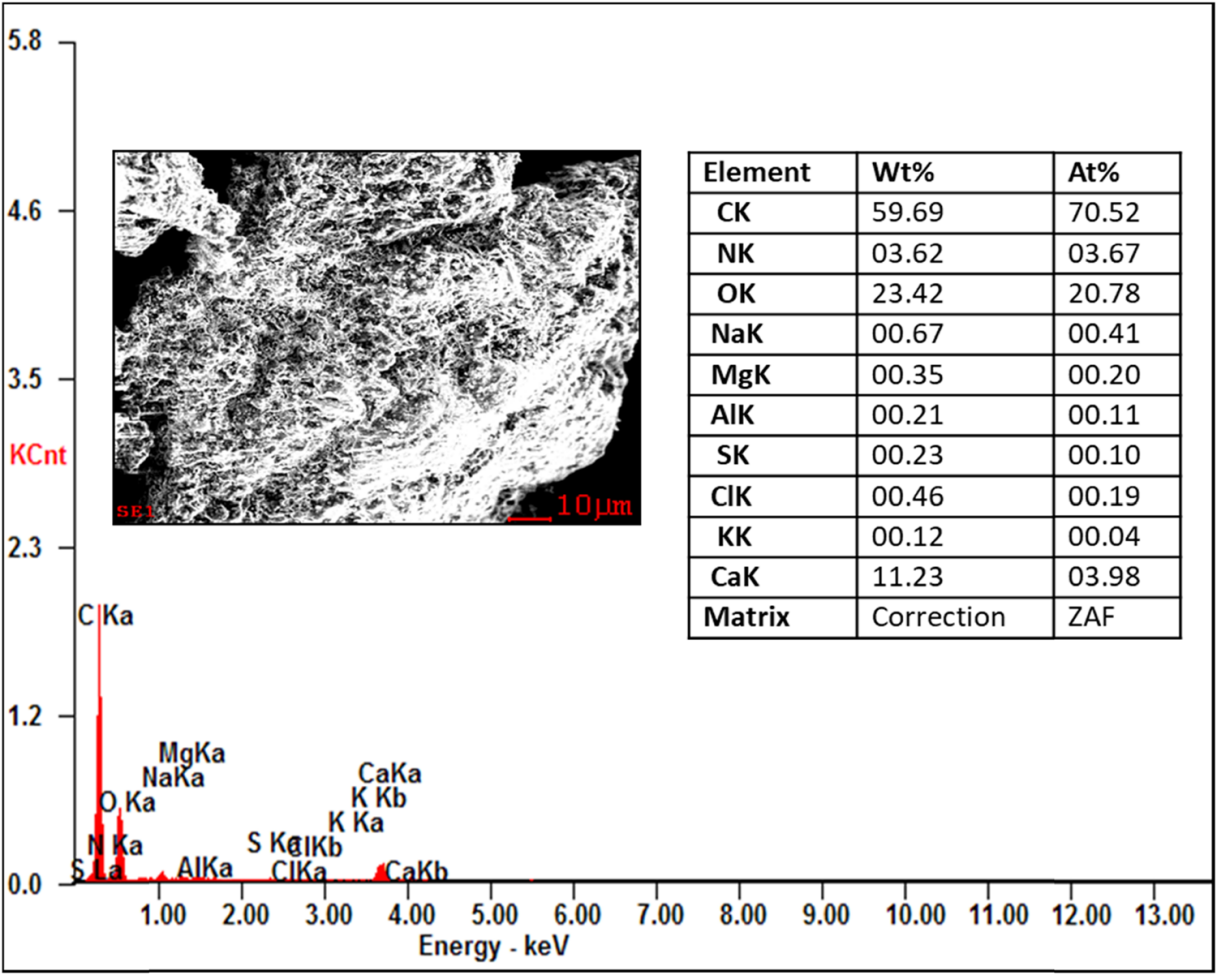
EDAX analysis of the raw fleshing sample.

**Figure 17:**
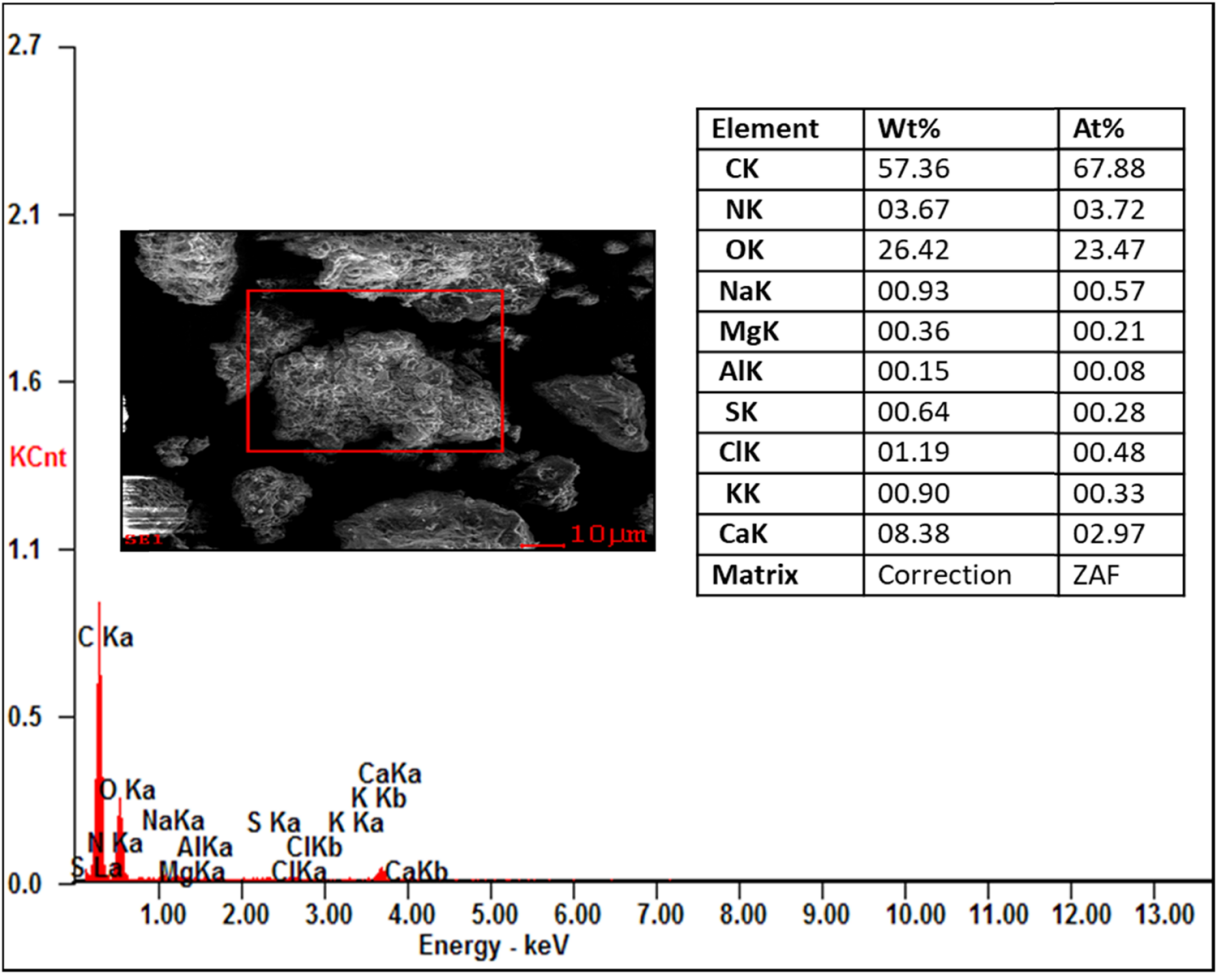
EDAX analysis of the digestate sample.

**Figure 18:**
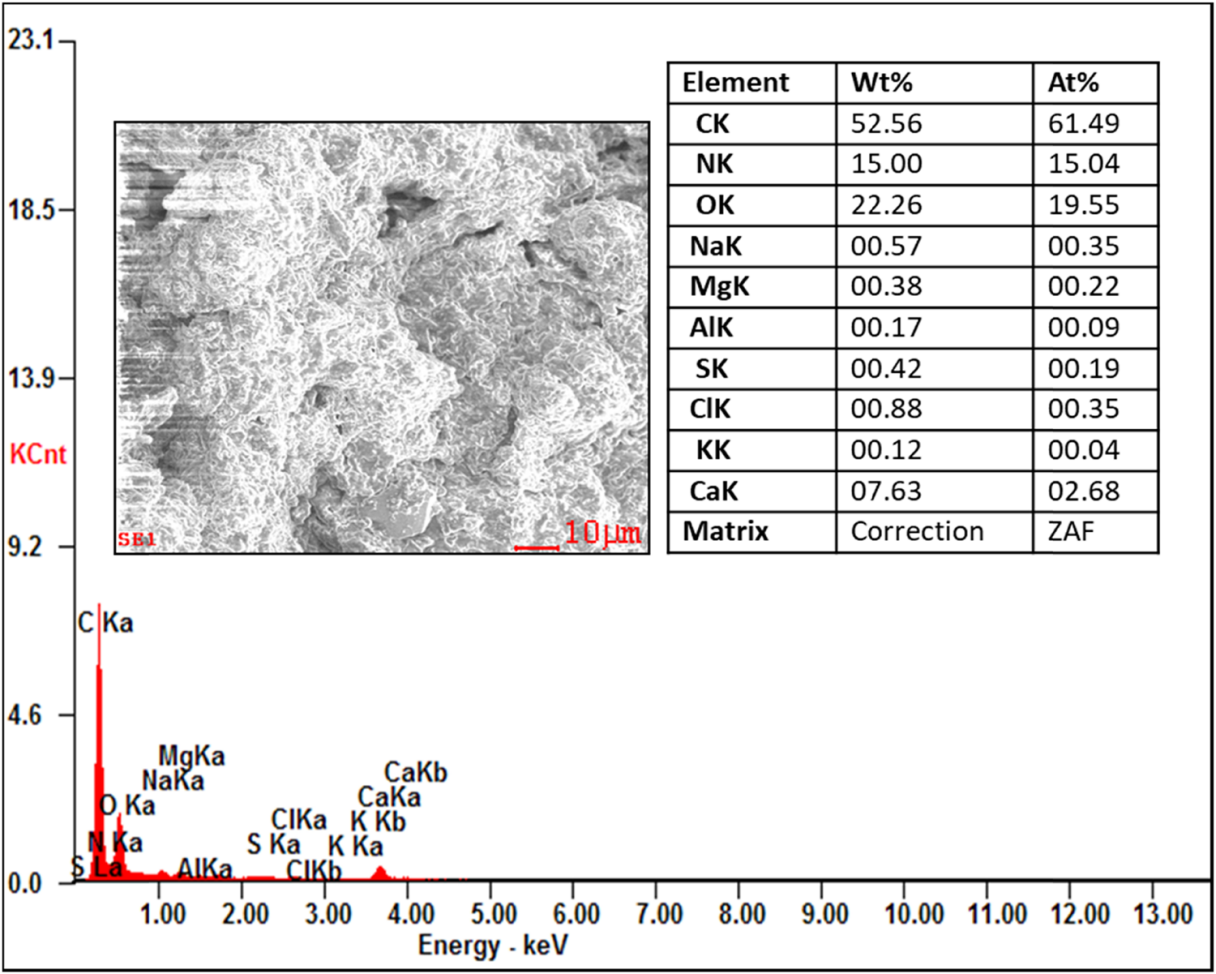
EDAX analysis of the trypsin-treated sample.

**Figure 19:**
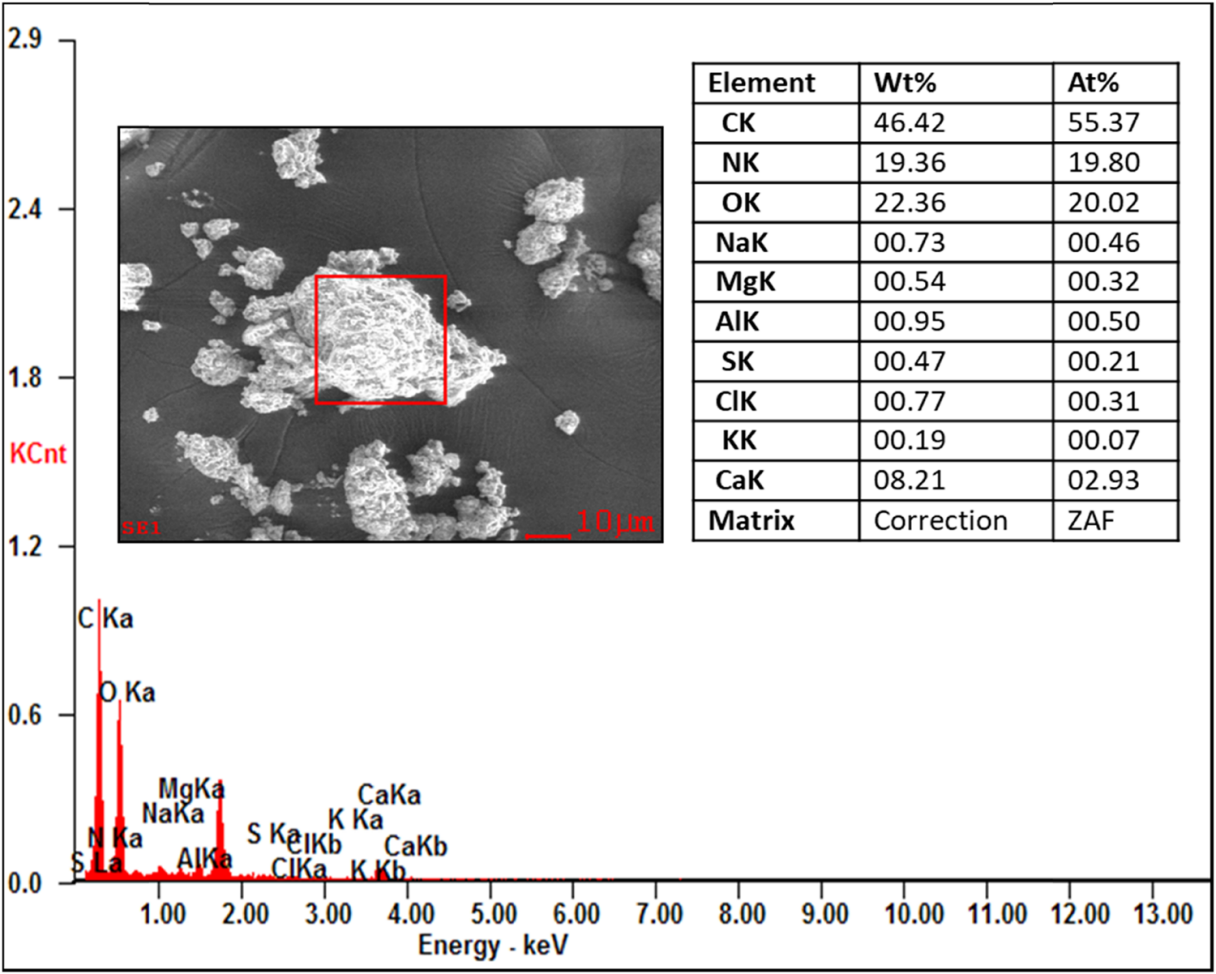
EDAX analysis of the papain-treated sample.

From the EDAX analysis, it is evident that there is not much variation in the chemical composition of organic and inorganic compounds like carbon (46.42–59.69%) and oxygen (22.26–26.42%). The nitrogen content of the proteolytic enzyme-treated samples showing the highest chemical composition (3.62–19.36%) compared to those of the raw and digestate samples. This increase in the nitrogen content is helpful in the alternation of the C/N ratio of the substrate for the process of AD to produce the biogas. The other compounds like sodium, aluminium, magnesium, sulphur, chlorine, potash, and calcium show the least in all the samples. No contamination peaks were observed in the samples, which represent the chemical purity of the samples. The presence of the compounds in the substrate is shown in Figures 16–19.

## 4 Conclusion

The pretreatment studies for tannery fleshings were carried out using proteolytic enzymes such as trypsin and papain for the liquefaction of fleshings with a 5U for the treatment of 1 kg fleshings. The batch scale experiment was carried out for a retention period of 45 days and the daily biogas was measured using the water displacement method. The maximum biogas of 12,118 mL from the standard BDS (25:75), 14073.25 mL from the trypsin-treated BDS (25:75), and 14881.25 mL from papain-treated BDS (25:75) were generated with methane contents of 63.77, 65.64, and 66.29%, respectively. There is an increase in the biogas production from 13.9 and 18.57% compared to standard BDS (25:75), which is not pretreated with enzyme. The kinetic models show the goodness of fit between 0.993 and 0.998. The correlation coefficient domain is [−1, 1] from a statistical perspective, which was observed in this work. The instrumental study of tannery fleshings concludes that TGA shows the weight loss of the sample with the thermal application for the analysis of thermal degradation of tannery fleshings from ambient temperature to 1,000°C. The different stages of sample degradation were observed from the TG and DTG profiles, which can be helpful for further thermochemical applications. The FTIR analysis shows the different functional groups stretching present in the samples at wavelengths between 400 and 4,000 cm^−1^, which confirms the presence of different functional groups in the tannery fleshings like –OH, –CO, –NH, –CH, N–O, NH_3_ with different peaks. Biodegradation of ketone and –C═O groups into new groups of acids was noticed. The synthesis of amino acid groups from these functional group degradations can speed up the hydrolysis process during the AD of tannery fleshings to create biogas. The SEM image revealed the presence of tiny fibrous tissue dispersion in the matrix. The fibrous protein was digested, as seen by the SEM image of the digestate and the disintegration occurred in the pretreated samples. Prior to digestion, a fine spread of fibrous tissues bound to proteins could be seen in SEM images; however, the fibrous structures were not present in the digestate. The identification of secondary metabolites produced during the digestive process is aided by instrumental analysis. The digestate has a rough and uneven surface with voids, which could be attributable to the emission of biogas during the digestion process. The EDAX analysis for the different samples shows variation in the chemical composition and also shows the increase of nitrogen in enzyme-treated samples, with carbon (46.42–59.69%) and oxygen (22.26–26.42%). The nitrogen content of the proteolytic enzyme treated showed the highest chemical composition (3.62–19.36%). The digestate of the AD process can be used as soil amendment for better crop yields.
